# Regional Validation and Recalibration of Clinical Predictive Models for Patients With Acute Heart Failure

**DOI:** 10.1161/JAHA.117.006121

**Published:** 2017-11-18

**Authors:** Benjamin S. Wessler, Robin Ruthazer, James E. Udelson, Mihai Gheorghiade, Faiez Zannad, Aldo Maggioni, Marvin A. Konstam, David M. Kent

**Affiliations:** ^1^ Tufts Cardiovascular Center Tufts Medical Center Boston MA; ^2^ Predictive Analytics and Comparative Effectiveness (PACE) Center Institute for Clinical Research and Health Policy Studies (ICRHPS) Tufts Medical Center/Tufts University School of Medicine Boston MA; ^3^ Northwestern University Feinberg School of Medicine Chicago IL; ^4^ Institut National de la Santé et de la Recherche Médicale (INSERM) Nancy France; ^5^ Associazione Nazionale Medici Cardioligi Ospedalieri Research Center Florence Italy

**Keywords:** acute heart failure, cardiovascular disease risk factors, clinical predictive model, external validation, modeling, prediction, prognostic factor, Heart Failure, Risk Factors, Quality and Outcomes

## Abstract

**Background:**

Heart failure clinical practice guidelines recommend applying validated clinical predictive models (CPMs) to support decision making. While CPMs are now widely available, the generalizability of heart failure CPMs is largely unknown.

**Methods and Results:**

We identified CPMs derived in North America that predict mortality for patients with acute heart failure and validated these models in different world regions to assess performance in a contemporary international clinical trial (N=4133) of patients with acute heart failure treated with guideline‐directed medical therapy. We performed independent external validations of 3 CPMs predicting in‐hospital mortality, 60‐day mortality, and 1‐year mortality, respectively. CPM discrimination decreased in all regional validation cohorts. The median change in area under the receiver operating curve was −0.09 (range −0.05 to −0.23). Regional calibration was highly variable (90th percentile of absolute difference between smoothed observed and predicted values range <1% to >50%). Calibration remained poor after global recalibrations; however, region‐specific recalibration procedures significantly improved regional performance (recalibrated 90th percentile of absolute difference range <1% to 5% across all regions and all models).

**Conclusions:**

Acute heart failure CPM discrimination and calibration vary substantially across different world regions; region‐specific (as opposed to global) recalibration techniques are needed to improve CPM calibration.

**Clinical Perspective**

**What Is New?**

- To assess the generalizability of acute heart failure clinical predictive models (CPMs), we validated and recalibrated a sample of acute heart failure CPMs predicting short‐ and long‐term mortality in different world regions.

**What Are the Clinical Implications?**

- CPM discrimination and calibration vary substantially across different world regions, and regional (as opposed to global) recalibration techniques were needed to improve CPM calibration.
- Off‐the‐shelf acute heart failure CPMs may support appropriate decision making in 1 region, while yielding misleading information in another.
- Region‐specific recalibrations can improve CPM calibration.

It is increasingly recognized that patients with the same disease can differ from one another substantially with respect to their outcome risks, and the harms and benefits of treatment.^1^, ^2^ To aid physicians and patients in individualizing decisions, clinical predictive models (CPMs) are now widely available to estimate the likelihood of important outcomes (prognostic models) or diagnoses (diagnostic models) based on patient‐specific characteristics.^3^ In the case of heart failure, CPMs have been proposed to inform decisions for advanced therapies and palliative care^4^ and also the common and costly admission decision for patients with acute heart failure (AHF) in the emergency department.^5^ While many different CPMs exist for predicting mortality for HF,^6^ CPM performance is often significantly better for the population on which the model was derived compared with similar yet distinct “validation” populations.^7^

Model performance across different world regions is largely unknown. Even within the restricted settings of randomized controlled trials for patients with HF, substantial regional heterogeneity in patient characteristics and in outcome rates have been observed.^8^, ^9^, ^10^ Thus, an important but understudied concern is that CPMs may support appropriate decision making in 1 region, while yielding misleading information in another. Here we use data from the EVEREST (Efficacy of Vasopressin Antagonism in Heart Failure Outcome Study with Tolvaptan) trial^11^ and perform regional independent external validations of previously published CPMs that predict mortality following hospital admission for AHF. We evaluate CPMs for AHF derived on data from patients in 1 world region (here, North America) and determine whether these CPMs can generalize to patients in different world regions (Eastern Europe, Western Europe, and South America and whether global or regional recalibration procedures improve regional performance.

## Methods

External validations explore CPM performance for patients not included in the derivation data set. The general approach requires matching CPMs to validation database(s) and assessing model performance. Here CPM performance was assessed in different world regions and recalibration techniques were evaluated.

### Model Selection

Identifying CPMs that match the validation database is a process that involves evaluation of both the original CPM and the validation cohorts (Table 1). For this analysis, “compatible CPMs” were defined by the following characteristics: (1) the index condition in the derivation cohort was similar to the index condition in the validation cohort (here AHF), (2) CPM predicts an outcome captured in the validation cohort (here mortality), (3) all variables in the CPM were captured in the validation data sets and can be assigned a value, and (4) CPMs were derived in patient samples from a single world region (here, North America). We identified compatible models by reviewing a recently published systematic review of CPMs for HF.^6^ For this analysis, we present a sample of the compatible CPMs developed in North America that predict mortality at 3 different time points (in‐hospital, 60 day, and 1 year) following hospitalization for HF.

**Table 1:** Baseline Characteristics for Patients Among the Various Databases

Variable	GWTG‐HF^a^	OPTIME‐CHF	EFFECT^a^	EVEREST	NA EVEREST	SA EVEREST	EE EVEREST	WE EVEREST
Years	2005–2007	1997–1999	1999–2001	2003–2006	2003–2006	2003–2006	2003–2006	2003–2006
Data source	Registry	Clinical trial	Clinical trial	Clinical trial	Clinical trial	Clinical trial	Clinical trial	Clinical trial
N	27 850	949	2624	4133	957	586	1552	477
Age	72.5^$^	68^&^	76.3^$^	67.0 (58.0–75.0)	70.0 (60.0–78.0)	63.0 (56.0–71.0)	66.0 (58.0–73.0)	70.0 (61.3–77.0)
SBP	137^&^	120^&^	148^$^	120.0 (105.0–131.0)	112.0 (101.0–128.0)	112.5 (100.0–117.1)	122.0 (110.0–140.0)	112.0 (100.0–130.0)
Na	138^&^	139^&^	138^$^	140.0 (137.0–142.0)	139.0 (136.0–142.0)	140.0 (137.0–142.0)	140.0 (138.0–143.0)	139.0 (137.0–142.0)
BUN, mg/dL	25^&^	13^&^	29.4^$^	26.0 (20.0–35.0)	30.0 (22.0–45.0)	25.00 (19.0–32.0)	23.0 (18.0–30.0)	31.0 (22.0–45.0)
Heart rate, BPM	82^&^	84^&^	94^$^	78.0 (69.0–90.0)	76.0 (68.0–86.0)	78.0 (69.5–90.0)	80.0 (70.0–90.0)	76.0 (68.0–88.0)
Respiratory rate	NR	NR	26^$^	20.0 (18.0–22.0)	20.0 (18.0–22.0)	20.0 (18.75–22.0)	20.0 (18.0–24.0)	20.0 (18.0–23.0)
Prior CVA, %	14	NR	17	17	28	13	16	15
COPD, %	28	23	21	10	18	6	5	9
Black race, %	18	33	NR	4	17	10	0	0
Hemoglobin	12.0^&^	NR	12.4^$^	13.2 (11.8–14.5)	12.5 (11.2–13.9)	13.5 (12.1–14.7)	13.7 (12.5–14.9)	13.0 (11.4–14.2)
NYHA class IV, %	NR	47	NR	42	44	46	43	34
Dementia, %	NR	b	9	b	b	b	b	b
Cancer, %	NR	b	9	b	b	b	b	b
Liver disease, %	NR	b	1	b	b	b	b	b

Clinical predictive models derivation populations are presented on the left (bold border). Validation data sets (overall and regional) are shown on the right. Gray shading indicates variables that are included in the CPM derived from each database. BUN indicates blood urea nitrogen; BPM, beats per minute; CVA, cerebrovascular accident; COPD, chronic obstructive pulmonary disease; CPM, clinical predictive model; EE, Eastern Europe; EFFECT, Enhanced Feedback for Effective Cardiac Treatment study; EVEREST, Efficacy of Vasopressin Antagonism in Heart Failure: Outcome Study with Tolvaptan; GWTG‐HF, Get With The Guidelines‐Heart Failure; NA, North American; NYHA, New York Heart Association; OPTIME‐CHF, The Outcomes of a Prospective Trial of Intravenous Milrinone for Exacerbations of Chronic Heart Failure study; SA, South America; SBP, systolic blood pressure; WE, Western Europe.

a Acute heart failure populations that include patients with both reduced and preserved ejection fractions.

b Variables that were exclusion criteria for a given database (these variables were coded as 0). NR indicates not reported. For the derivation populations, continuous variables are shown as means ($) or medians (&) as originally presented. For the validation populations, values are presented as median (interquartile range).

### Selected Models

Selected validated models are shown in Table 1 and Figure S1. Selected models were as follows: GWTG‐HF^12^ (The American Heart Association Get With the Guidelines‐Heart Failure) model (7 variables, predicts in‐hospital mortality), OPTIME‐CHF^13^ (Outcomes of a Prospective Trial of Intravenous Milrinone for Exacerbations of Chronic Heart Failure) (5 variables, predicts 60‐day mortality after admission), and EFFECT^14^ (Enhanced Feedback for Effective Cardiac Treatment) model (10 variables, predicts 1‐year mortality after admission).

The GWTG‐HF program collected patient‐level data from patients hospitalized for HF at 287 hospitals in the United States between January 2005 and June 2007.^12^ These data were used to build and validate a model predicting in‐hospital mortality following admission for HF that was presented as a point score and online calculator in 2010. The model was built using logistic regression analysis from a final cohort of 27 850 patients (derivation cohort) and validated on 11 933 patients (validation cohort) from this program. It has since been externally validated.^15^

The OPTIME‐CHF study was a randomized clinical trial of 949 patients with HF with reduced ejection fraction hospitalized for worsening symptoms.^16^ Patients were randomized to receive intravenous milrinone or placebo for 48 to 72 hours. The outcome of 60‐day mortality did not differ significantly between the milrinone and placebo groups (10.3% versus 8.9%, *P*=0.41). Patients were enrolled from 78 centers across the United States from 1997 to 1999. A CPM based on a point score predicting 60‐day mortality was derived from this data set using Cox proportional hazards analysis and internally validated in this database.^13^

The EFFECT study group presented a CPM derived from 2624 patients hospitalized in Ontario, Canada, from April 1999 to March 2001 for HF. Data for this model came from the Canadian Institutes of Health Information hospital discharge abstract and patients were included only if they met a prespecified definition of clinical HF. This CPM was created using logistic regression analysis and validated on 1407 patients from different hospitals in Ontario from a previous time period (1997–1999).

### External Validation Cohort

**Figure 1:**
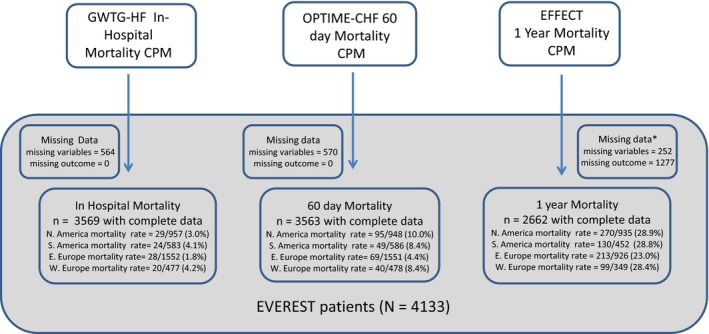
GWTG‐HF is Get with the Guidelines‐Heart Failure in‐hospital mortality CPM. OPTIME‐CHF is Outcomes of a Prospective Trial of Intravenous Milrinone for Exacerbations of Chronic Heart Failure 60‐d mortality CPM. EFFECT is the Enhanced Feedback for Effective Cardiac Treatment 1‐y mortality CPM. Validation exercises were done for patients with all variables available. *Indicates that for the 1‐y mortality model, we considered patients to have missing data if they were last known alive with <9 mo of follow‐up. CPM indicates clinical predictive models.

The EVEREST trial has been previously reported.^17^ This was a prospective, international, randomized, placebo‐controlled study conducted in 359 sites worldwide from 2003 and 2006. The trial included 1251 patients from North America, 699 patients from South America, 564 patients from Western Europe, and 1619 patients from Eastern Europe (Figure 1). This study evaluated the addition of tolvaptan to standard medical therapy for AHF and reduced ejection fraction and enrolled patients within 48 hours of HF hospitalization. During a median follow‐up of 9.9 months, 537 (26%) of the patients died and tolvaptan had no effect on long‐term mortality for these patients (hazard ratio 0.98; 95% confidence interval, 0.87%–1.11%; *P*=0.68). The patients enrolled in this trial were treated with guideline‐directed medical therapies for HF including angiotensin‐converting enzyme inhibitors (84%), β‐blockers (70%), aldosterone blockers (54%), and diuretics (97%) and thus this trial provides an opportunity to evaluate the regional performance of previously published CPMs on an international population of patients with AHF treated with contemporary evidence‐based therapies.

### Outcomes

All models were tested for their ability to predict all‐cause mortality in the overall EVEREST cohort and separately in regional EVEREST cohorts using patient‐level data. The GWTG‐HF in‐hospital mortality model was validated on in‐hospital mortality in the EVEREST study; the OPTIME‐CHF 60‐day mortality model was validated on 60‐day mortality in the EVEREST study; the EFFECT study 1‐year mortality model was validated on 1‐year mortality in the EVEREST study (Figure 1). Patients censored prior to 1 year were either dropped from the analysis (if last known alive and followed for <9 months, n=1471) or included as alive (if alive and followed for ≥9 months, n=2662). Sensitivity analyses to explore these assumptions are presented in Figure S2A through S2D.

### Statistical Analysis and Model Recalibration

Our approach to validating these CPMs used patient‐level data from EVEREST. For each patient and each CPM we calculated a point score based on covariate values. This point score was then converted into predicted event probabilities as described by the original CPM authors (Figure S1). When a range of probabilities was given, the midpoint probability was assigned for a given point score range. For various performance measures and both global and regional recalibration procedures, the estimated event probabilities were converted to the linear predictor using the equation [predicted value=(1/(1+e^−xbeta^))] where xbeta is the linear predictor. We evaluated the loss in discrimination by assessing the change in Area under the Receiver Operating Curve (AUC). Percent decrement in discrimination was calculated as [Derivation AUC−0.5]−[Regional AUC−0.5]/[Derivation AUC−0.5]×100. All analyses were run in R Studio Version 0.99.489.

### Measuring CPM Performance

*Calibration‐in‐the‐large* is a measure of global fit. *Model discrimination* was represented here by the AUC. In this analysis, we assess percent decrement in discrimination, which is derived from the AUC for each region. *Model calibration* was assessed primarily through calibration plots. We also report Harrell's E statistic, which calculates a prediction error for each individual patient by using a lowess‐estimated probability as the observed outcome rate.^18^ We report E_90_ and E_avg_ statistics in this report. E_avg_ computes the average absolute calibration error (average absolute difference between the lowess‐estimated calibration curve and the line of identity). E_90_ describes the 90th percentile of the absolute differences (ie, 90% of individuals have absolute prediction errors that are below this value).

### Recalibration

CPM recalibration techniques have been previously described.^19^ The simplest form of recalibration (technique 1) addresses calibration‐in‐the‐large and considers the mean observed outcome rate in the derivation and validation cohorts and applies the difference between these rates to update the intercept (α) of the CPM. The next form of recalibration (technique 2) adjusts both the intercept and the slope (ie, applies a uniform correction factor to the regression coefficients of the independent variables to better fit the validation population). This recalibration technique corrects both for differences in prevalence unrelated to covariate effects (as in technique 1) and also can correct for overfitting in the derivation population. To assess whether global or region‐specific recalibrations are needed to improve CPM performance, our recalibrations proceeded stepwise, first with global recalibrations on the entire EVEREST cohort (techniques 1 and 2) and next with region‐specific recalibrations (techniques 1 and 2).

This study was reviewed and approved via expedited review procedures by the Tufts Health Sciences IRB and informed consent requirement was waived.

## Results

The covariates that are used to calculate probabilities with each CPM are shown in Table 1. Overall the patients in the derivation cohorts appear similar (related) to the patients in the validation cohorts (EVEREST database overall and region specific). The distribution of covariates is shown for each world region within the validation databases. The numbers of cases with complete data and the number of outcomes for each time point and each region are shown in Figure 1. Two CPMs (GWTG‐HF and EFFECT) were derived from data sets including both patients with HF with reduced ejection fraction and those with preserved ejection fraction. GWTG‐HF CPM was derived from registry data. The OPTIME‐CHF CPM was derived from data collected between 5 and 7 years before the EVEREST study was conducted. Exclusion criteria for these databases are shown in Table S1. The randomized controlled trials had more exclusion criteria than the registry database.

### Independent External Validations

CPM discrimination was assessed across different world regions, and we observed major decrements in the ability of the CPMs to discriminate between those who died from those who did not (Table 2). Even within the North American EVEREST cohort, there was a substantial decrement in model discrimination, with percent decrement ranging from −19% for the EFFECT CPM predicting 1‐year mortality to −30% for the OPTIME‐CHF model predicting 60‐day mortality. The median model percent decrement in discrimination across all world regions and all CPMs was −35%. The median percent decrement in discrimination for GWTG‐HF CPM was −42% and in South America the CPM had essentially no ability to effectively rank event probabilities (AUC 0.54). The median percent decrement in discrimination for OPTIME‐CHF CPM was 26% with the worst performance in Western Europe (AUC 0.66). The EFFECT CPM had a median percent decrement in discrimination of 43% and had the poorest discrimination in South America (AUC 0.58).

**Table 2:** Discrimination

CPM	Derivation AUC	Worldwide AUC [95% CI] (% Decrement)	North America AUC [95% CI] (% Decrement)	South America AUC [95% CI] (% Decrement)	Eastern Europe AUC [95% CI] (% Decrement)	Western Europe AUC [95% CI] (% Decrement)
GWTG‐HF	0.75	0.64 [0.60–0.69] (−44%)	0.70 [0.62–0.77] (−20%)	0.54 [0.42–0.66] (−84%)	0.65 [0.58–0.73] (−40%)	0.64 [0.55–0.74] (−44%)
OPTIME‐CHF	0.77	0.72 [0.68–0.75] (−19%)	0.69 [0.64–0.74] (−30%)	0.69 [0.61–0.77] (−30%)	0.71 [0.64–0.78] (−22%)	0.66 [0.57–0.74] (−41%)
EFFECT	0.77	0.66 [0.64–0.68] (−41%)	0.72 [0.68–0.75] (−19%)	0.58 [0.53–0.64] (−70%)	0.62 [0.58–0.66] (−56%)	0.69 [0.58–0.66] (−30%)

AUC indicates area under the receiver operator curve, % decrement is the percent decrease in discrimination and is calculated as [Derivation AUC−0.5]−[Regional AUC−0.5]/[Derivation AUC−0.5]×100; CI, confidence interval; CPM, clinical predictive models; EFFECT, Enhanced Feedback for Effective Cardiac Treatment study; GWTG‐HF, Get With The Guidelines‐Heart Failure; OPTIME‐CHF, Outcomes of a Prospective Trial of Intravenous Milrinone for Exacerbations of Chronic Heart Failure study.

We assessed calibration‐in‐the‐large for each mortality time point (in‐hospital mortality, 60‐day mortality, and 1‐year mortality) for the validation databases (Table 3). The in‐hospital mortality rate was 2.8% in the EVEREST trial. GWTG‐HF CPM had excellent calibration‐in‐the‐large for Eastern Europe and North America, while substantially underpredicting overall event rates in South America and Western Europe (difference in observed versus predicted event rates is −2.1% and −1.7%, respectively). The 60‐day mortality rate in the EVEREST trial was 7.1%. OPTIME‐CHF CPM predicted 60‐day mortality rates were considerably higher than observed rates; the difference in observed versus predicted event rates ranged from 8.3% in Eastern Europe to 19.2% in North America. By 1 year, 26.7% of patients in the overall EVEREST trial had died. The EFFECT CPM systematically underpredicted overall 1‐year event rates across the different world regions, particularly in Eastern Europe and South America (by −5.0% and −9.1%, respectively).

**Table 3:** Calibration‐in‐the‐Large

Model	Event Rate	EVEREST	N. America	S. America	E. Europe	W. Europe
GWTG‐HF (in hospital)	Observed event rate	0.028	0.030	0.041	0.018	0.042
	Average Pred. rate	0.022 (0.016)	0.027 (0.021)	0.020 (0.014)	0.017 (0.012)	0.025 (0.018)
	Diff. (Obs.−Pred.)	0.006	0.003	0.021	0.001	0.017
OPTIME‐CHF (60 d)	Observed event rate	0.071	0.100	0.084	0.045	0.084
	Average Pred. rate	0.198 (0.223)	0.292 (0.258)	0.172 (0.192)	0.128 (0.166)	0.271 (0.25)
	Diff. (Obs.−Pred.)	−0.127	−0.192	−0.088	−0.083	−0.187
EFFECT (1 y)	Observed event rate	0.267	0.289	0.288	0.230	0.283
	Average Pred. rate	0.227 (0.152)	0.271 (0.169)	0.197 (0.131)	0.180 (0.115)	0.274 (0.170)
	Diff. (Obs.−Pred.)	0.040	0.018	0.091	0.050	0.009

Observed and Predicted average event rates in the validation data sets. Average Pred. Rate indicates the mean predicted outcome rates in the validation data sets (SD); Diff. (Obs.−Pred.), the difference between the Observed event rate and the average predicted event rate; E. Europe, Eastern European patients in EVEREST; EVEREST, Efficacy of Vasopressin Antagonism in Heart Failure: Outcome Study with Tolvaptan; GWTG‐HF, Get With The Guidelines‐Heart Failure; N. America, North American patients in EVEREST; S. America, South American patients in EVEREST; W. Europe, Western European patients in EVEREST.

**Figure 2:**
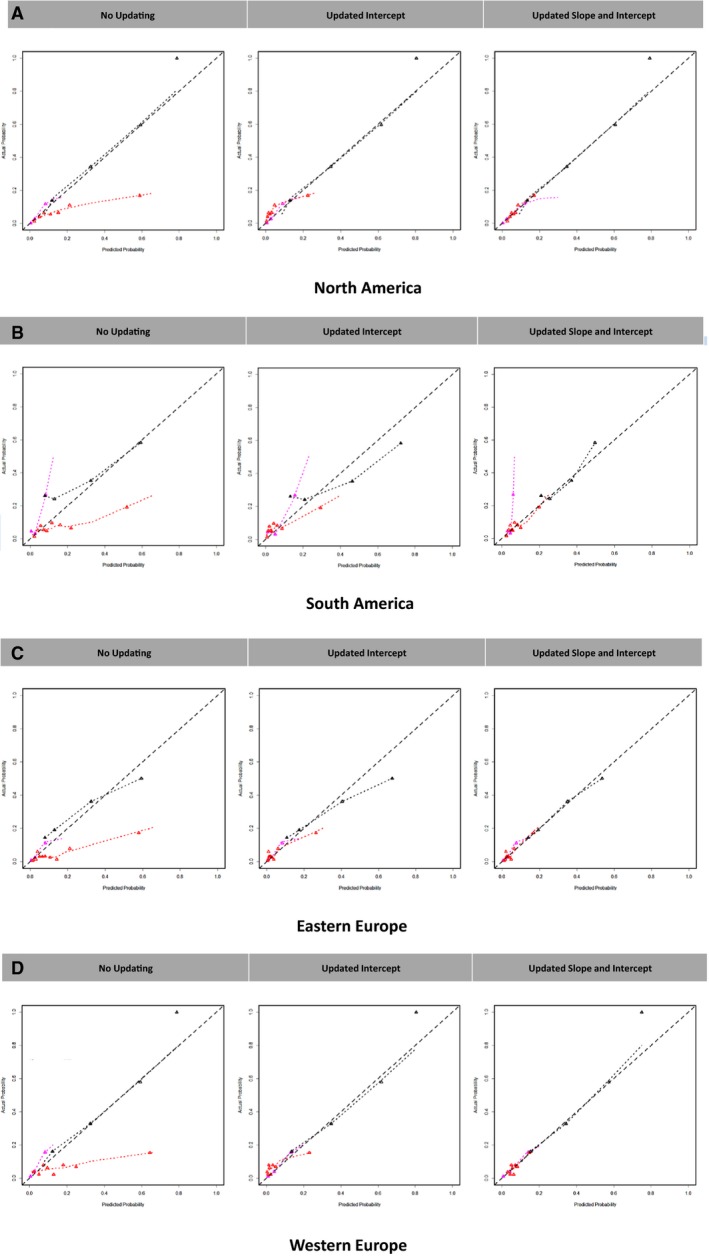
GWTG‐HF is Get With the Guidelines–Heart Failure in‐hospital mortality CPM. OPTIME‐CHF is Outcomes of a Prospective Trial of Intravenous Milrinone for Exacerbations of Chronic Heart Failure 60‐d mortality CPM. EFFECT is the Enhanced Feedback for Effective Cardiac Treatment 1‐y mortality CPM. No updating is the original CPM applied to the validation population. Updated intercept is technique 1 with regional updating, Updated Intercept and Slope is technique 2 with regional updating (described in the text). A, North American calibration plots, (B) South American calibration plots, (C) Eastern European calibration plots, (D) Western European calibration plots. Calibration plots are presented according to deciles of predicted probabilities. CPM indicates clinical predictive models.

We assessed model calibration across ranges of predicted risk for different world regions. Regional calibration plots (without recalibration) are shown in Figure 2A through 2D. These curves demonstrate highly variable and generally poor calibration. For the GWTG‐HF CPM without recalibration the E_90_ ranged from <1% in Eastern Europe and North America to 3.9% in South America. The OPTIME‐CHF CPM demonstrated substantial miscalibration with the E_90_ ranging from 19% in Eastern Europe to 51% in Western Europe. For the EFFECT CPM, calibration varied significantly across different world regions where the E_90_ ranged from 3% in North America to 18% in South America. Tables S2 and S3 show a summary of CPM calibration across the different regional validation populations.

### Model Recalibration (Global)

Our first set of recalibrations was based on global adjustments of the intercept (technique 1) and intercept and slope (technique 2), (Table S3). Despite global recalibration of the intercept, GWTG‐HF CPM predicting in‐hospital mortality E_90_ remained at 3.8% in South America, OPTIME‐CHF CPM predicting 60‐day mortality remained poorly calibrated in certain regions (eg, E_90_ was 13.7% in Western Europe) and the EFFECT CPM predicting 1‐year mortality showed only minimal improvement from baseline performance (recalibrated E_90_ ranged from 4.4% to 16.1% across different world regions). Recalibrations based on global adjustment of the intercept and slope (technique 2) yielded similar results. GWTG‐HF CPM E_90_ ranged from <1% to 3.7%, OPTIME‐CHF CPM remained poorly calibrated (eg, E_90_ was 7.5% in South America), and EFFECT CPM predicting 1‐year mortality also showed only minimal improvement from the base model performance (recalibrated E_90_ ranged from 1.1% to 12.9% across different world regions).

### Model Recalibration (Regional)

Next we applied technique 1 using region‐specific recalibrations (Figure 2A through 2D and Table S3). Despite region‐specific updating of the intercept, the regional calibration of the GWTG‐HF CPM predicting in‐hospital mortality remained essentially unchanged (E_90_ ranged from <1% to 3.4% across different world regions). Technique 1 regional recalibration led to only modest improvements in regional calibration for the OPTIME‐CHF CPM predicting 60‐day mortality, and miscalibration for this CPM was most significant in South America where E_90_ remained at 13.5%. Following technique 1 recalibration, the regional calibration for the EFFECT CPM predicting 1‐year mortality showed only minimal improvement (E_90_ was 12.9% in South America).

Regional recalibration of the CPM intercept and slope (technique 2) demonstrated significant improvements in calibration (Figure 2A through 2D and Table S3). Following technique 2 recalibration, E_90_ for the GWTG‐HF CPM predicting in‐hospital mortality decreased to ≤1.4% across all world regions. This regional recalibration technique lowered E_90_ for the OMPTIME‐CHF CPM predicting 60‐day mortality and the EFFECT CPM predicting 1‐year mortality across all world regions to ≤2.2% and ≤5.1%, respectively. The region‐specific intercept and slope corrections that optimize calibration are shown in Table S2. In general, the OPTIME‐CHF CPM and the EFFECT CPM had recalibrated slopes that were <1 across all world regions, suggesting that the original models were substantially overfit. Notably, the major decrements in discrimination that we observed remain unchanged despite the various recalibration procedures.

## Discussion

Here a series of independent external validations demonstrate that published CPMs for AHF frequently perform poorly (with respect to discrimination and calibration) and have limited generalizability. Further, performance can vary substantially across different world regions even in the same clinical trial with uniform inclusion criteria. Finally, performance (specifically calibration) can be improved significantly with simple recalibration procedures, but only when recalibration is performed using region‐specific corrections. Since different adjustments (to intercept and slope) are necessary to optimize performance across various world regions, it appears unrealistic to expect a single “off‐the‐shelf” CPM to perform well across all settings.

Consistent with a recent report limited only to North America,^15^ The GWTG‐HF CPM showed a moderate drop in discrimination in our North American validation cohort. CPM discrimination across different world regions was generally considerably worse for each of the 3 models compared with performance reported in the initial derivation samples and the decrement in discrimination varied substantially across different world regions. This may reflect (1) overfitting in the derivation population; (2) differences in case‐mix/disease severity across regions; and (3) phenotype heterogeneity across regions (ie, the effects of the independent variables may be different across the different populations). Techniques that minimize the risks of overfitting include avoiding data‐driven variable selection procedures and ensuring a large number (*often between 10 and 20*) events per considered variable.^20^, ^21^ An example of this heterogeneity is noted in South America where the causes of HF are different and also use of certain therapies (such as implantable cardioverter‐defibrillators and β‐blockers) are less common.^8^ While the percent decrement in discrimination in different world regions is often large, we acknowledge uncertainty surrounding these point estimates. Unfortunately, the simple recalibration techniques done here (in the absence of adding variables or recalculating individual beta coefficients) do nothing to improve this loss of discrimination.

A similarly important (and often neglected^22^) measure of performance is calibration. Calibration of the originally published CPMs varies across world regions and is often poor. The reasons for poor regional calibration include regional differences in HF causes, severity, and treatment.^8^, ^23^, ^24^ Additionally, certain variables such as New York Heart Association class^25^ and various vital signs^26^ are likely captured with varying fidelity across different databases and regions. It is also likely that the threshold to admit patients for AHF, local systems for postdischarge care, and follow‐up are all highly variable across the globe and relate to prognosis. Reasonable local calibration is essential since applying poorly calibrated models to inform clinical decisions—such as discharging low‐risk patients from the hospital or considering advanced therapies for high‐risk patients—holds the potential to do harm when compared with “treat all” or “treat none” approaches. Good calibration protects models from motivating harmful changes in decisions regardless of model discrimination.^27^, ^28^

Simple recalibration techniques can significantly improve calibration, and the recalibration procedures needed to optimize performance are region specific. As CPMs are used to aid clinical decisions, it is important to understand model performance within local care systems. If models are used for administrative purposes, differences between observed and predicted event rates related to processes of care (and not poor CPM performance) may be informative and potentially actionable. Without these independent external measures of performance, our assessment of CPMs (and the information they yield) is incomplete (at best) and potentially harmful.

Our study had several limitations. First, our sample of AHF models did not comprehensively explore all published AHF CPMs and may not be representative of models generally or HF models in particular. We believe that these models are representative of AHF CPMs generally since they were created from contemporary clinical trial and registry data, have been variably incorporated into guidelines, and have been previously validated by the original investigators. There are certain validation data sets in specific regions with modest size (≈400 patients) and also low event rates (≈2.5% for in‐hospital mortality). These characteristics may adversely affect our ability to measure CPM performance.^27^ The GWTG‐HF and EFFECT were derived on patients with AHF and preserved and reduced ejection fraction while the EVEREST database included only a subset of these patients (with reduced ejection fraction). If the effects of covariates are different across these unique HF subtypes or if there is less relatedness between these populations, then we should anticipate worse model performance across the EVEREST databases. Also, the CPMs examined here were point scores with predications based on *observed* outcome rates in point score strata rather than model‐based probability estimates. Using these observed rates may have increased the error in prediction. Nevertheless, these observed outcome rates are presented in the original CPM articles as substitutes for risk predictions, and so are appropriate to use in our analysis. Finally, we used complete case analyses in these validations, which may bias our results if the included cases are not representative of the larger population of patients with AHF. This is unlikely to be a major concern since the patients included in the complete case analyses of these CPMs appear very similar across the different analytic timeframes (Table S4).

Performance of these North American CPMs for AHF is generally poor and varies substantially across different world regions. Simple recalibration procedures improve the calibration (but not discrimination) of previously published CPMs for regional populations with AHF, but only when region‐specific recalibrations are applied. This analysis shows the importance of independent external validations, especially when clinical decisions might be leveraged by the output. Poorly calibrated models hold the potential for harm and there should be renewed emphasis on *local* performance of CPMs.

## Sources of Funding

This work was partially supported through a Patient‐Centered Outcomes Research Institute (PCORI) Methods Award (ME‐1606‐35555), as well as by the National Institutes of Health (T32 HL069770 Training Grant from the NIH, 5 TL1 TR001062 Training Grant from the NIH‐NCATS, 4U01NS086294‐04). All statements in this report, including its findings and conclusions, are solely those of the authors and do not necessarily represent the views of the PCORI, its Board of Governors, or Methodology Committee.

## Disclosures

Drs Udelson, Konstam, Zannad, and Gheorghiade received research support from Otsuka for participating in the original EVEREST trial. The current analysis was not funded by Otsuka.

## Supporting information


**Table S1.** Database Exclusion Criteria
**Table S2.** Regional Intercept and Slope Corrections
**Table S3.** Calibration With Various Recalibration Techniques
**Table S4.** Comparison Included Versus Excluded
**Figure S1.** Originally Presented Point Scores described by the authors. These predictive models allow for calculation of individual event rates based on clinical variables.
**Figure S2.** A, Sensitivity analysis of EFFECT CPM. Including only patients dead or alive with >12 mo of follow‐up. B, Sensitivity analysis of EFFECT CPM. Including only patients dead or alive with >6 mo of follow‐up. C, Sensitivity analysis of EFFECT CPM. Including only patients dead or alive with >9 mo of follow‐up. D, Sensitivity analysis of EFFECT CPM. Patient's status alive or dead imputed according to survival probability at last follow‐up n=3881.
