# Supplementary material for: Regional Validation and Recalibration of Clinical Predictive Models for Patients With Acute Heart Failure
Source: J Am Heart Assoc. 2017 Nov 18;6(11):e006121. doi: 10.1161/JAHA.117.006121 (PMC5721739; doi:10.1161/JAHA.117.006121)

SUPPLEMENTAL MATERIAL

Table S1. Database Exclusion Criteria

Database	Exclusion Criteria
**OPTIME CHF**	1. Patient requires IV vasopressor or inotropic support. 2. Patient requires admission primarily for concurrent morbidity. Left ventricular failure primarily from uncorrected obstructive valvular disease, hypertrophic obstructive cardiomyopathy, uncorrected thyroid disease, known acute myocarditis, known amyloid cardiomyopathy, or known malfunctioning artificial heart valve. 4. Patient is scheduled for heart surgery. 5. There is evidence of unstable angina, active myocardial ischemia, or myocardial infarction within 3 months. 6. Patient has atrial fibrillation with a sustained ventricular response rate >110 beats/min. 7. Patient has sustained ventricular tachycardia or fibrillation. 8. Patient has systolic blood pressure <80 or >150 mm Hg. 9. Patient has severe renal impairment with a creatinine level >3.0 mg/dL or requires dialysis. 10. Patient has suspected digitalis intoxication. 11. Patient has known hypersensitivity to milrinone.
**EFFECT**	Patients who developed heart failure after admission (ie, in-hospital complication), patients transferred from another acute care facility, those aged 105 years or older, nonresidents, and those with an invalid health card
**GWTG-HF**	Patients were excluded from analysis if they did not have a diagnosis of HF, if they were transferred to a different acute care facility, if the discharge date was invalid, or if data were missing for their discharge status , or left ventricular ejection fraction (LVEF).
**EVEREST**	Cardiac surgery within 60 d of potential study enrollment, excluding percutaneous coronary interventions. Planned revascularization procedures, electrophysiologic device implantation, cardiac mechanical support implantation, cardiac transplantation, or other cardiac surgery within 30 days after study enrollment. Subjects who are on cardiac mechanical support. History of biventricular pacer placement within the last 60 d. Comorbid condition with an expected survival < 6 mo. Subjects with acute ST segment elevation myocardial infarction at the time of hospitalization. History of sustained ventricular tachycardia or ventricular fibrillation within 30 days, unless in the presence of an automatic implantable cardioverter defibrillator. History of a cerebrovascular accident within the last 30 d. Hemodynamically significant uncorrected primary cardiac valvular disease. Hypertrophic cardiomyopathy (obstructive or nonobstructive) Congestive heart failure from uncorrected thyroid disease, active myocarditis, or known amyloid cardiomyopathy. Subjects with refractory, end-stage, heart failure defined as subjects who are appropriate candidates for specialized treatment strategies, such as ventricular assist devices, continuous positive intravenous inotropic therapy, or hospice care Progressive or episodic neurologic disease such as multiple sclerosis or history of multiple Strokes. History of primary significant liver disease or acute hepatic failure. Chronic uncontrolled diabetes mellitus as determined by the investigator. Subjects currently treated

	with hemofiltration or dialysis. Morbid obesity, defined as 159 kg (or 350 lb) or body mass index 42. Supine systolic arterial blood pressure 90 mm Hg. Serum creatinine 3.5 mg/dL or 309.4 µmol/L. Serum potassium 5.5 mEq/L or 5.5 mmol/L. Hemoglobin 9 g/dL or 90 g/L or 5.586 mmol/L. History of hypersensitivity or idiosyncratic reaction to benzazepine derivatives (such as benazepril). Women who will not adhere to the reproductive precautions as outlined in the informed consent form. Positive urine pregnancy test. Inability to provide written informed consent. History of drug or medication abuse within the past year, or current alcohol abuse. Previous participation in this or any other tolvaptan clinical trial. Inability to take oral medications. Participation in another clinical drug or device trial in which the last dose of drug was within the past 30 d or an investigation medical device is implanted

Exclusion criteria as written in the original reports (Person et al, Lee et al.) or the Design and Rationale resports (Cuffe et al. and Gheorghiade et al)

Table S2. Regional Intercept and Slope Corrections

Model	Timeframe	Intercept, slope (Worldwide)	Intercept, slope (North America)	Intercept, slope (South America)	Intercept, slope (Eastern Europe)	Intercept, slope (Western Europe)
GWTG-HF	In hospital	-0.159, 0.883	1.21, 1.335	-2.783, 0.099	-0.318, 0.917	0.748, 1.061
OPTIME-CHF	60 days	-1.806, 0.532	-1.777, 0.468	-1.482, 0.558	-1.849, 0.626	-1.983, 0.375
EFFECT	1 year	-0.028, 0.753	0.070, 0.965	-0.190, 0.461	-0.118, 0.687	-0.025, 0.854

Optimized regional intercept and slope corrections that optimize calibration so that predicted outcome rates match observed outcome rates.

Table S3. Calibration with Various Recalibration Techniques

	Model	Recalibration method	Eavg (E90) North America	Eavg (E90) South America	Eavg (E90) Eastern Europe	Eavg (E90) Western Europe
*Regional Calibration without updating	GWTG-HF	None	0.004 (0.005)	0.021 (0.039)	0.001 (0.001)	0.017 (0.014)
	OPTIME-CHF	None	0.193 (0.478)	0.092 (0.395)	0.084 (0.185)	0.192 (0.505)
	EFFECT	None	0.022 (0.030)	0.095 (0.182)	0.058 (0.065)	0.020 (0.040)
# Regional Calibration with various Global Recalibration techniques	GWTG-HF	Intercept	0.008 (0.007)	0.017 (0.038)	0.005 (0.008)	0.009 (0.006)
		Slope and Intercept	0.009 (0.008)	0.017 (0.037)	0.006 (0.008)	0.009 (0.006)
	OPTIME	Intercept	0.055 (0.110)	0.031 (0.042)	0.017 (0.018)	0.058 (0.137)
		Slope and Intercept	0.010 (0.019)	0.018 (0.075)	0.011 (0.024)	0.015 (0.035)
	EFFECT	Intercept	0.028 (0.047)	0.079 (0.161)	0.034 (0.044)	0.034 (0.063)
		Slope and Intercept	0.031 (0.066)	0.051 (0.129)	0.006 (0.011)	0.025 (0.031)
¥Calibration with various Regional Recalibration techniques.	GWTG-HF	Intercept	0.005 (0.006)	0.027 (0.034)	0.002 (0.001)	0.004 (0.003)
		Slope and Intercept	0.002 (0.003)	0.019 (0.014)	0.001 (0.001)	0.004 (0.004)
	OPTIME-CHF	Intercept	0.049 (0.079)	0.037 (0.135)	0.018 (0.016)	0.048 (0.084)
		Slope and Intercept	0.007 (0.012)	0.009 (0.022)	0.006 (0.015)	0.005 (0.006)
	EFFECT	Intercept	0.013 (0.019)	0.073 (0.129)	0.031 (0.044)	0.024 (0.028)
		Slope and Intercept	0.010 (0.014)	0.025 (0.051)	0.006 (0.012)	0.012 (0.016)

*represents regional calibration without recalibration. # represents regional calibration with Global recalibrations.¥represents regional calibration with region specific recalibrations. GWTG-HF predicts in-hospital mortality. OPTIME-CHF predicts 60 day mortality, EFFECT predicts 1 year mortality. Recalibration method is the technique of model updating. Intercept is update of the intercept to the overall database for the global recalibrations and to the specific region for the regional recalibrations. , Slope and Intercept is update of the slope and intercept to the overall database for the global recalibrations and to the specific region for the regional recalibrations. Eavg is Harrell's E statistic and represents the average difference between observed and predicted values. E90 represents the 90th percentile of absolute difference between observed and predicted values.

Table S4. Comparison Included vs. Excluded

Data source and Variable	Pooled	Include	Exclude	p-value	test
**I. GWTIn (In-Hospital Outcome Model)**	**N=4133**	**N=3568 (86%)**	**N=565 (13%)**		
Age	65.8 +/- 11.9 (4133)	65.8 +/- 11.8 (3568)	65.2 +/- 12.0 (565)	0.2486	(ttest)
Systolic blood pressure	120.5 +/- 19.7 (4091)	120.7 +/- 19.7 (3568)	118.8 +/- 19.1 (523)	0.0392	(ttest)
Sodium	139.6 +/- 4.6 (4030)	139.7 +/- 4.7 (3568)	139.2 +/- 4.0 (462)	0.0520	(ttest)
Blood urea nitrogen	30.2 +/- 16.3 (3960)	30.2 +/- 16.1 (3568)	30.3 +/- 18.4 (392)	0.9044	(ttest)
Death outcome_in hosp	2.6% (109/4129)	2.8% (101/3568)	1.4% (8/561)	0.0537	
region	N=4133	N=3568	N=565	<.0001	(chisq) df=3
EASTERN EUROPE	39.2% (1619)	43.5% (1552)	11.9% (67)		
NORTH AMERICA	30.3% (1251)	26.8% (956)	52.2% (295)		
SOUTH AMERICA	16.9% (699)	16.3% (583)	20.5% (116)		
WESTERN EUROPE	13.6% (564)	13.4% (477)	15.4% (87)		
**II. Optime60 (60day Outcome Model)**	**N=4133**	**N=3569 (86%)**	**N=564 (13%)**		
Age , mean +/- stdev	65.8 +/- 11.9 (4133)	65.8 +/- 11.8 (3563)	65.3 +/- 12.2 (570)	0.3302	(ttest)
Systolic blood pressure	120.5 +/- 19.7 (4091)	120.7 +/- 19.7 (3563)	118.9 +/- 19.1 (528)	0.0489	(ttest)
Sodium	139.6 +/- 4.6 (4030)	139.7 +/- 4.7 (3563)	139.2 +/- 4.0 (467)	0.0326	(ttest)
Blood urea nitrogen	30.2 +/- 16.3 (3960)	30.1 +/- 16.1 (3563)	30.5 +/- 18.6 (397)	0.6340	(ttest)
Death outcome_60d	7.1% (295/4133)	7.1% (253/3563)	7.4% (42/570)	0.8177	(chisq) df=1
region	N=4133	N=3563	N=570	<.0001	(chisq) df=3
EASTERN EUROPE	39.2% (1619)	43.5% (1551)	11.9% (68)		
NORTH AMERICA	30.3% (1251)	26.6% (948)	53.2% (303)		
SOUTH AMERICA	16.9% (699)	16.4% (586)	19.8% (113)		
WESTERN EUROPE	13.6% (564)	13.4% (478)	15.1% (86)		
**IIIb. Effect365 (365 day Outcome Model)**	**N=4133**	**N=2662 (64%)**	**N=1471 (36%)**		
Age	65.8 +/- 11.9 (4133)	65.8 +/- 12.2 (2662)	65.6 +/- 11.3 (1471)	0.6335	(ttest)
Systolic blood pressure	120.5 +/- 19.7 (4091)	119.4 +/- 19.6 (2662)	122.5 +/- 19.6 (1429)	<.0001	(ttest)
Sodium	139.6 +/- 4.6 (4030)	139.4 +/- 4.8 (2658)	140.0 +/- 4.3 (1372)	0.0001	(ttest)
Blood urea nitrogen	30.2 +/- 16.3 (3960)	31.1 +/- 17.1 (2662)	28.2 +/- 14.4 (1298)	<.0001	(ttest)
Death outcome_ 1 year	26.8% (765/2856)	26.7% (712/2662)	27.3% (53/194)	0.8619	
region	N=4133	N=2662	N=1471	<.0001	(chisq) df=3
EASTERN EUROPE	39.2% (1619)	34.8% (926)	47.1% (693)		
NORTH AMERICA	30.3% (1251)	35.1% (935)	21.5% (316)		
SOUTH AMERICA	16.9% (699)	17.0% (452)	16.8% (247)		
WESTERN EUROPE	13.6% (564)	13.1% (349)	14.6% (215)		

Figure S1. Originally Presented Point Scores described by the authors. These Predictive Models allow for calculation of individual event rates based on clinical variables.

Systolic BP	Points	BUN	Points	Sodium	Points	Age	Points
50-59	28	≤9	0	≤130	4	≤19	0
60-69	26	10-19	2	131	3	20-29	3
70-79	24	20-29	4	132	3	30-39	6
80-89	23	30-39	6	133	3	40-49	8
90-99	21	40-49	8	134	2	50-59	11
100-109	19	50-59	9	135	2	60-69	14
110-119	17	60-69	11	136	2	70-79	17
120-129	15	70-79	13	137	1	80-89	19
130-139	13	80-89	15	138	1	90-99	22
140-149	11	90-99	17	≥139	0	100-109	25
150-159	9	100-109	19			≥110	28
160-169	8	110-119	21				
170-179	6	120-129	23				
180-189	4	130-139	25				
190-199	2	140-149	27				
≥200	0	≥150	28				

Heart Rate	Points	Black Race	Points	COPD	Points	Total Score	Probability of Death
≤79	0	Yes	0	Yes	2	**0-33**	**<1%**
80-84	1	No	3	No	0	**34-50**	**1-5%**
85-89	3					**51-57**	**>5-10%**
90-94	4					**58-61**	**>10-15%**
95-99	5					**62-65**	**>15-20%**
100-104	6					**66-70**	**>20-30%**
≥105	8					**71-74**	**>30-40%**
						**75-78**	**>40-50%**
						**≥79**	**>50%**

Reproduced with permission from: Peterson PN, Rumsfeld JS, Liang L, Albert NM, Hernandez AF, Peterson ED, Fonarow GC, Masoudi F a. A validated risk score for in-hospital mortality in patients with heart failure from the American Heart Association get with the guidelines program. *Circ Cardiovasc Qual Outcomes*. 2010;3:25–32.

Table 5. Nomogram for Predicting 60-Day Mortality in Decompensated Heart Failure

Age	Points	Sodium	Points	NYHA Class IV	Points
20	0	115	79	No	0
30	8	120	69	Yes	23
40	17	125	59		
50	25	130	49		
60	33	135	30		
70	41	140	20		
80	50	145	10		
90	58	150	0		

SBP	Points	BUN	Points
80	94	5	10
90	86	10	20
100	77	15	30
110	69	20	40
120	60	25	50
130	51	30	60
140	43	35	70
150	34	40	80
160	26	45	90
170	17	50	100
180	9		
190	0		

Total points	Predicted 60-day mortality
124	2%
149	4%
163	6%
174	8%
182	10%
208	20%
225	30%

Reproduced with permission from: Felker GM, Leimberger JD, Califf RM, Cuffe MS, Massie BM, Adams KF, Gheorghiade M, O'Connor CM. Risk stratification after hospitalization for decompensated heart failure. *J Card Fail*. 2004;10:460–466.

Table 4. Heart Failure Risk Scoring System*

Variable	No. of Points: 30-Day Score†	No. of Points: 1-Year Score‡
Age, y	+Age (in years)	+Age (in years)
Respiratory rate, min (minimal 20; maximum 45)§	+Rate (in breaths/min)	+Rate (in breaths/min)
Systolic blood pressure, mm Hg‖		
≥180	−60	−50
160-179	−55	−45
140-159	−50	−40
120-139	−45	−35
100-119	−40	−30
90-99	−35	−25
<90	−30	−20
Urea nitrogen (maximum, 60 mg/dL)§¶	+Level (in mg/dL)	+Level (in mg/dL)
Sodium concentration <136 mEq/L	+10	+10
Cerebrovascular disease	+10	+10
Dementia	+20	+15
Chronic obstructive pulmonary disease	+10	+10
Hepatic cirrhosis	+25	+35
Cancer	+15	+15
Hemoglobin <10.0 g/dL (<100 g/L)	NA	+10

Abbreviation: NA, not applicable to 30-day model.
*An electronic version of the risk scoring system is available at: http://www.ccort.ca/CHFriskmodel.asp.
†Calculated as age + respiratory rate + systolic blood pressure + urea nitrogen + sodium points + cerebrovascular disease points + dementia points + chronic obstructive pulmonary disease points + hepatic cirrhosis points + cancer points.
‡Calculated as age + respiratory rate + systolic blood pressure + urea nitrogen + sodium points + cerebrovascular disease points + dementia points + chronic obstructive pulmonary disease points + hepatic cirrhosis points + cancer points + hemoglobin points.
§Values higher than maximum or lower than minimum are assigned the listed maximum or minimum values.
‖Increases were protective in both mortality models. Points are subtracted for higher blood pressure measurements.
¶Maximum value is equivalent to 21 mmol/L. Score calculated using value in mg/dL.

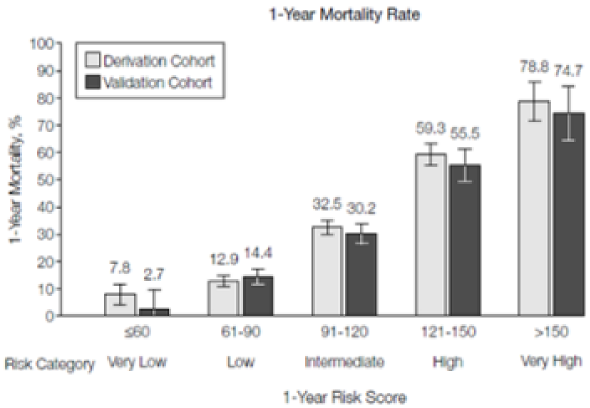

Reproduced with permission from: Lee DS, Austin PC, Rouleau JL, Liu PP, Naimark D, Tu J V. Predicting mortality among patients hospitalized for heart failure: derivation and validation of a clinical model. *JAMA*. 2003;290:2581–7.

Figure S2a. Sensitivity Analysis of EFFECT CPM
Including only patients dead or alive with > 12 months of follow up

Region	AUC
EVEREST	0.66
North America	0.71
South America	0.59
Eastern Europe	0.62
Western Europe	0.68

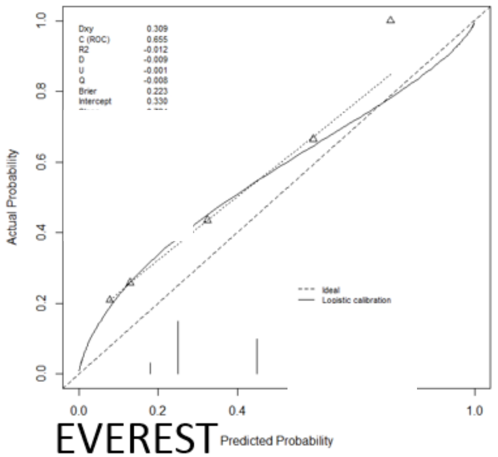

EVEREST

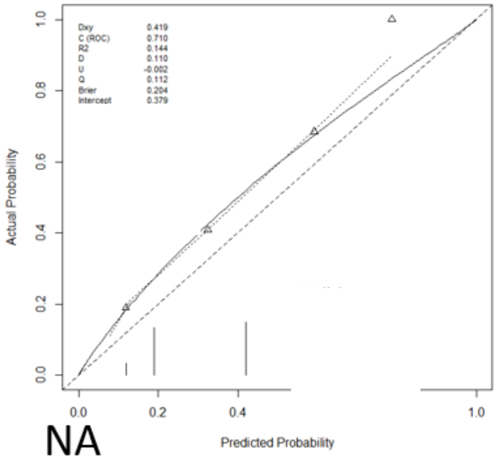

NA

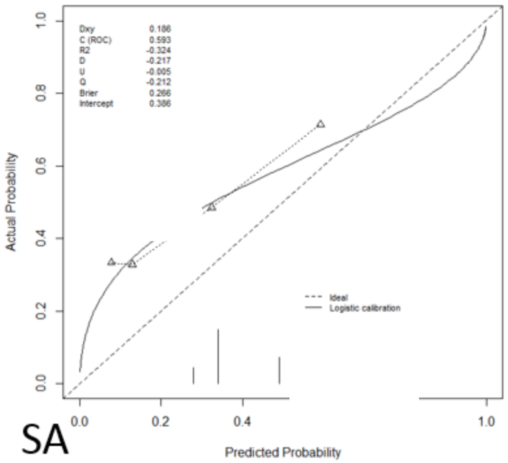

SA

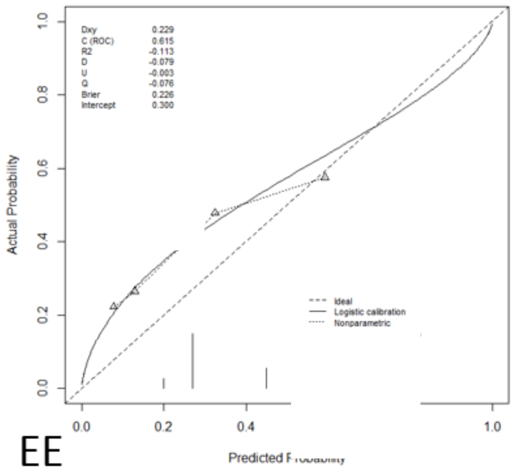

EE

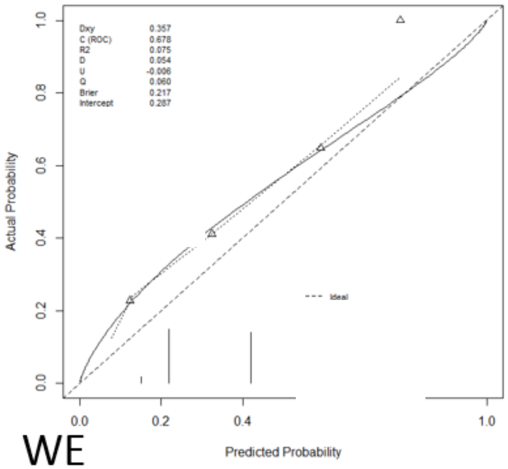

WE

Figure S2b. Sensitivity Analysis of EFFECT CPM. Including only patients dead or alive with ≥ 6 months of follow up

Region	AUC
EVEREST	0.68
North America	0.73
South America	0.58
Eastern Europe	0.64
Western Europe	0.71

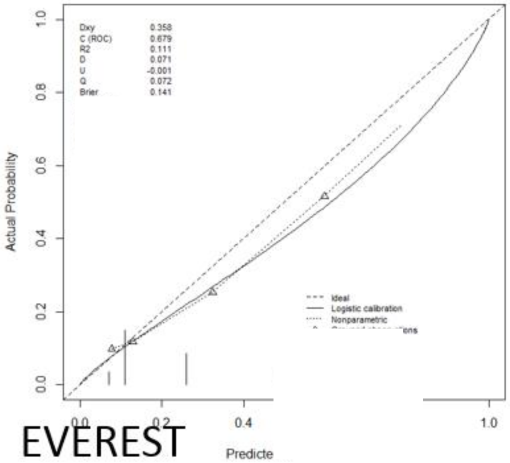

EVEREST

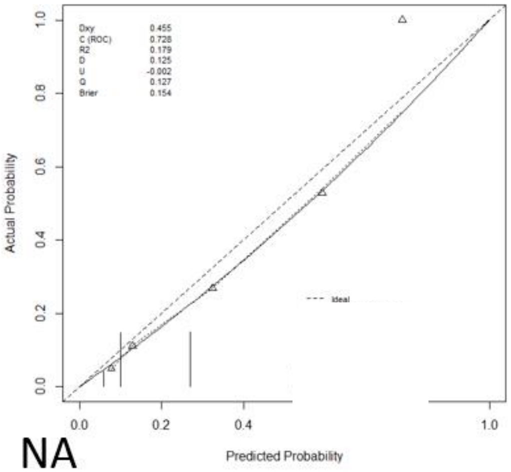

NA

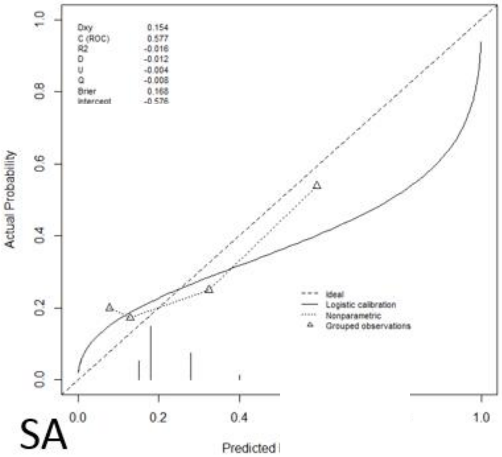

SA

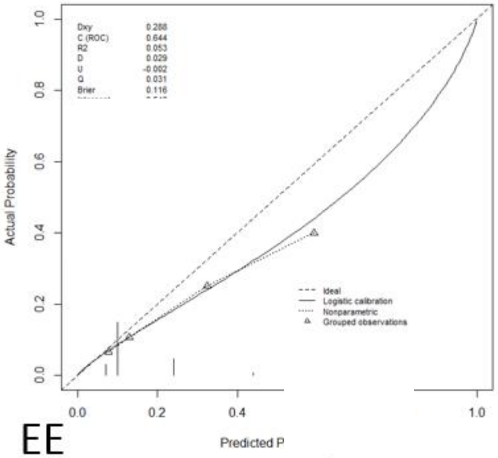

EE

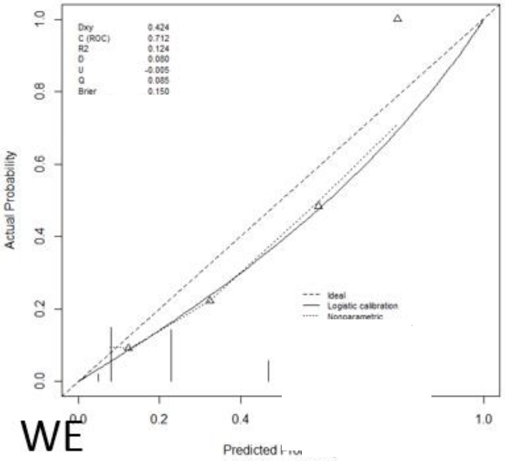

WE

Figure S2c. Sensitivity Analysis of EFFECT CPM (Including only patients dead or alive with > 9 months of follow up

Region	AUC
EVEREST	0.66
North America	0.72
South America	0.58
Eastern Europe	0.62
Western Europe	0.69

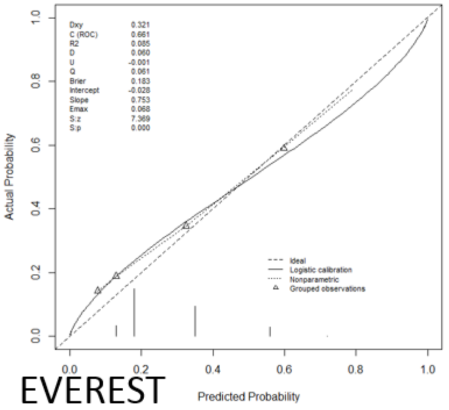

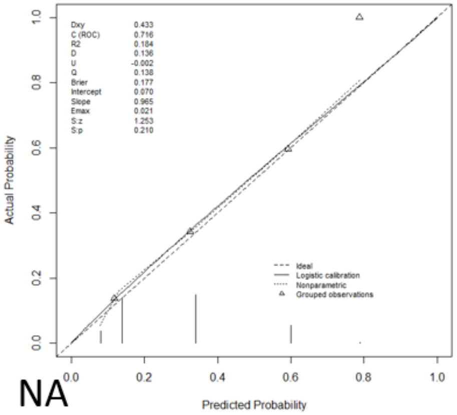

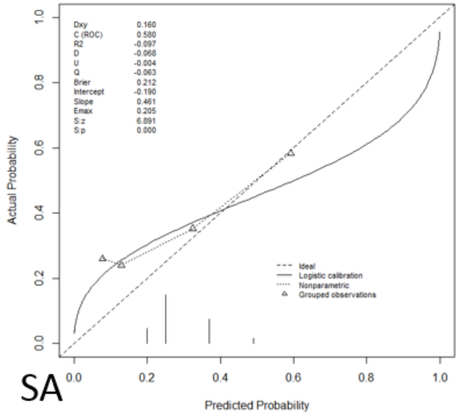

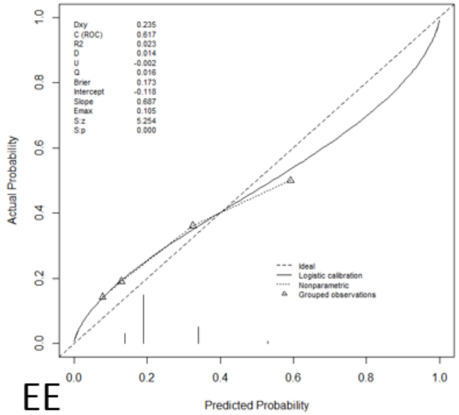

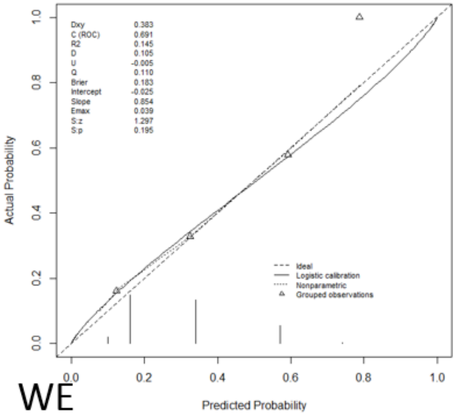

Figure S2d. Sensitivity Analysis of EFFECT CPM
Patient’s status alive or dead imputed according to survival probability at last follow up n = 3881

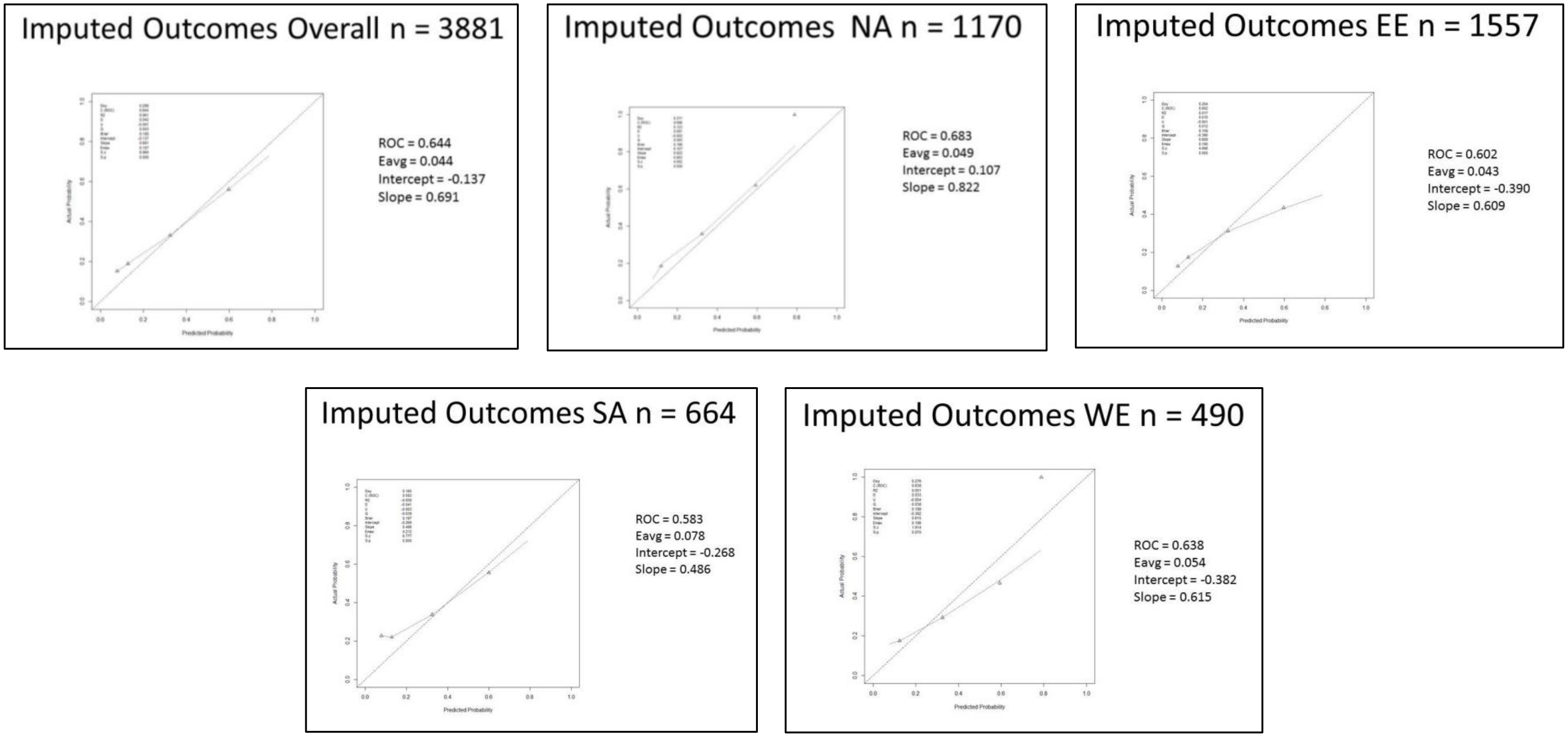

Supplement: Supplementary file 1 — Table S1. Database Exclusion Criteria Table S2. Regional Intercept and Slope Corrections Table S3. Calibration With Various Recalibration Techniques Table S4. Comparison Included Versus Excluded Figure S1. Originally Presented Point Scores described by the authors. These predictive models allow for calculation of individual event rates based on clinical variables. Figure S2. A, Sensitivity analysis of EFFECT CPM. Including only patients dead or alive with >12 mo of follow‐up. B, Sensitivity analysis of EFFECT CPM. Including only patients dead or alive with >6 mo of follow‐up. C, Sensitivity analysis of EFFECT CPM. Including only patients dead or alive with >9 mo of follow‐up. D, Sensitivity analysis of EFFECT CPM. Patient's status alive or dead imputed according to survival probability at last follow‐up n=3881. [file JAH3-6-e006121-s001.pdf]
